# Persistent, new-onset symptoms and mental health complaints in Long COVID in a Brazilian cohort of non-hospitalized patients

**DOI:** 10.1186/s12879-022-07065-3

**Published:** 2022-02-08

**Authors:** Ricardo Titze-de-Almeida, Thaylise Ramalho da Cunha, Letícia Dias dos Santos Silva, Clarisse Santos Ferreira, Caroline Pena Silva, Adriana Pinheiro Ribeiro, Agenor de Castro Moreira Santos Júnior, Pedro Renato de Paula Brandão, Andrezza Paula Brito Silva, Márcia Cristina Oliveira da Rocha, Mary-Ann Elvina Xavier, Simoneide Souza Titze-de-Almeida, Helena Eri Shimizu, Raimundo Nonato Delgado-Rodrigues

**Affiliations:** 1 Research Center for Major Themes, University of Brasília-Central Institute of Sciences, Brasília, Brazil; 2 Armed Forces Hospital, Learning and Research Technical Board, Brasília, Brazil; 3 Central Laboratory of Public Health of the Federal District, Brasília, Brazil; 4 Federal District Strategic Health Management Institute, Brasília, Brazil; 5 Department of Collective Health, Research Center for Major Themes, University of Brasília, Brasília, Brazil; 6 Central Institute of Sciences, Technology for Gene Therapy Laboratory-FAV, University of Brasília, Brasília, Brazil

**Keywords:** COVID-19, Long COVID, Memory, Sleep, Depression, Brazil

## Abstract

**Background:**

Severe acute respiratory syndrome coronavirus 2 (SARS-CoV-2) infections lead to acute- and chronic Long COVID (LC) symptoms. However, few studies have addressed LC sequelae on brain functions. This study was aimed to examine if acute symptoms of coronavirus disease 2019 (COVID-19) would persist during LC, and if memory problems would be correlated with sleep, depressive mood, or anxious complaints.

**Methods:**

Our work followed a cohort of 236 patients from two public hospitals of the Federal District in mid-western Brazil. Patients’ interviews checked for clinical symptoms during acute and LC (5–8 months after real-time reverse transcription polymerase chain reaction, RT-qPCR).

**Results:**

Most cases were non-hospitalized individuals (86.3%) with a median age of 41.2 years. While myalgia (50%), hyposmia (48.3%), and dysgeusia (45.8%) were prevalent symptoms in acute phase, fatigue (21.6%) followed by headache (19.1%) and myalgia (16.1%) commonly occurred during LC. In LC, 39.8% of individuals reported memory complaints, 36.9% felt anxious, 44.9% felt depressed, and 45.8% had sleep problems. Furthermore, memory complaints were associated with sleep problems (adjusted OR 3.206; 95% CI 1.723–6.030) and depressive feelings (adjusted OR 3.981; 95% CI 2.068–7.815).

**Conclusions:**

The SARS-CoV-2 infection leads to persistent symptoms during LC, in which memory problems may be associated with sleep and depressive complaints.

**Supplementary Information:**

The online version contains supplementary material available at 10.1186/s12879-022-07065-3.

## Background

Coronavirus disease 2019 (COVID-19) is characterized by a severe acute respiratory syndrome due to coronavirus 2 (SARS-CoV-2) [1, 2]. Since the first cases detected in China, the infection numbers have rapidly grown. Up to date, over 251 million cases were confirmed globally, with ≈21.9 million registered in Brazil, where death number has surpassed 610 thousand (until November 12, 2021) [3]. Beyond actions for controlling the virus spreading and lethality, the long-term sequelae of COVID-19 need to be better characterized in Brazil. Generally, long COVID (LC) is defined by the persistence of COVID-19 symptoms or their relapse after weeks or months from the initial infection, independent of the SARS-CoV-2 presence as real-time reverse transcription polymerase chain reaction PCR (RT-qPCR) results are negative in many cases [4]. The literature considers 2 different phases: (a) post-acute (3 weeks to 3 months after the initial symptoms) and (b) chronic (after 3 months) [5].

A previous study about LC in non-hospitalized patients revealed that two-thirds of non-critically infected individuals still present anosmia/ageusia, dyspnea, or asthenia after two months of disease onset [6–8]. On top of these common symptoms were deterioration in mental health and quality of life [9]. In Brazil, a cross-sectional health survey demonstrated that the levels of depression (40%), anxiety (52%), and the onset of sleep problems (43%) increased during this public calamity [8] when compared to previously reported prevalence rates [10]. Changes in sleep and lifestyle influence our mental health and stress response. Additionally, SARS-CoV-2 may directly affect the brain through immune mechanisms [11], triggering neuropsychiatric disorders or altering cognitive function and mental health. Considering the high rates of SARS-CoV-2 infection in Brazil, dimensioning the impact of LC in the population is a crucial public health issue.

Currently, the testing and treatment of COVID-19 depend heavily on the Brazilian ‘Sistema Único de Saúde—SUS’ (Unified Health System), a health care system of the federal government that represents the only option of public assistance for 70% of the population. In Brazil, to test for SARS-CoV-2 infection using the RT-qPCR, one must present COVID-19 symptoms and a medical request. These criteria were followed by the two public hospitals of the present study, which provided a suitable setting for studying acute and LC symptoms in RT-qPCR positive cases. The present study was aimed to examine whether acute symptoms of COVID-19 would persist for up to 5–8 months at the LC phase in mainly non-hospitalized patients from mid-western Brazil. This study also evaluated if memory problems reported during LC would be associated with sleep, depressive mood, and anxious complaints.

## Methods

This longitudinal cohort study included patients from the ‘Hospital Regional de Santa Maria’ (HRSM) and the ‘Hospital de Base do DF’ (HBDF) in the Federal District, Brazil. Between September and December 2020, consecutive adult patients clinically diagnosed with COVID-19 and confirmed by RT-qPCR testing from nasopharyngeal swab, performed at ‘Laboratório Central de Saúde Pública do Distrito Federal’ (LACEN-DF), were invited to participate. All patients that accepted signed an informed consent form. Ethics Committee approval was granted by the Institute of Strategic Health Management of the Federal District (IGESDF), with CAEE number 36147920.1.0000.8153.

The training process for collecting data was done to obtain the most accurate information available. Firstly, all researchers completed the online course ‘COVID-19 contact tracing’ (https://www.coursera.org/learn/covid-19-contact-tracing). Then, they were carefully trained and supervised by senior researchers, tutors, and physicians for conducting interviews by phone calls and collect data from enrolled participants.

This study was divided into two time frames considering the RT-qPCR test date. The first one evaluated data from the first two weeks after a COVID-19 RT-qPCR positive test, named ‘acute phase’. The second time frame was named ‘Long COVID (LC)’ and occurred 5–8 months after RT-qPCR test positivity, corresponding to the chronic COVID-19. Our study set this 4-month period of 5–8 months after diagnosis, considering that it would represent the clinical presentation of chronic LC from two public hospitals in the Federal District. According to current literature, the chronic LC phase can develop after 3 months of disease onset [4, 5]. All demographic characteristics and clinical data for acute and chronic phases were obtained via phone calls, using structured questionnaires to assess typical symptoms of COVID-19 and LC complaints regarding sleep, depression, memory, comorbidities, and other data, as described in appendix 1 and 2 in Additional file 1. Anxiety data was collected with the Generalized Anxiety Disorder 2-item questionnaire (GAD-2) ≧ 3 points [12–14]. Researchers used the ‘TeamDesk’ platform (https://www.teamdesk.net) to manage the collected data. This SQL (Structured Query Language) relational database designed for the project uses a virtual cloud and backups of cryptographed information. Deceased, unreachable, and bedridden were excluded.

### Statistical analysis

Continuous variables were expressed as mean and standard deviation and categorical variables as the number of occurrences and percentages. Some variables were organized into categories, such as age (18–29, 30–49, 50–64, and older than 64 years) and the body mass index (BMI) (underweight, normal, overweight, and obese) [15]. The total number of reported symptoms from acute and LC phases were categorized in the following groups: 1–2, 3–5, 6–8, and 9–12. Individuals referring no symptoms were classified as asymptomatic. Symptoms during the LC phase were classified as ‘persistent’ if manifested by the same patient during both the acute and chronic LC phase or ‘new-onset symptom’ if the symptom was absent at the acute phase but manifested at the chronic LC phase 5–8 months after diagnosis. We used multiple logistic regression to estimate the odds ratio (OR) and 95% confidence intervals (CIs) for association between the outcome ‘memory complaint’ and the phenotypes of ‘sleep problems’, ‘depression’, and ‘anxiety’. Statistical analyses were carried out using Prism 9 software (Version 9.1.2). *P* < 0.05 was considered statistically significant.

## Results

Our cohort included 362 participants from HRSM and HBDF hospitals, with COVID-19 diagnosis confirmed by RT-qPCR and eligible for the follow-up at 5–8 months after diagnosis. Among these individuals, 71 (19.6%) were not reached, and 55 (15.2%) did not want to participate in this follow-up phase. In total, 236 (65.2%) individuals consented to participate in both study phases. Demographic data and clinical characteristics of the patients are described in Table 1. The cohort consisted predominantly of non-hospitalized patients (n = 201; 86.3%). Among patients who required hospitalization, 32 (13.7%) were at the COVID-19 hospital ward, and only 8 (3.4%) required critical care at the intensive care unit (ICU).

**Table 1:** Demographic and clinical features of COVID-19 positive cases from HRSM and HB Brazilian hospitals (n = 236)

	Values
Woman, n (%)	144 (61.0%)
Age, mean (SD), range, y	41.2 (12.8) 19.0–81.0
Age distribution, n (%)	
18–29 y	46 (19.5%)
30–49 y	129 (54.7%)
50–64 y	48 (20.3%)
> 64 y	13 (5.5%)
Body mass index (BMI), mean (SD), range, kg/m^2^	27.8 (5.7) 17.1–51.3
WHO BMI classification, n (%)*	
Underweight	4 (1.8%)
Normal	63 (28.8%)
Overweight	86 (39.3%)
Obese	61 (27.9%)
Number of reported symptoms during acute infection phase, mean (SD), range	4.1 (3.0) 0–12
Number of reported symptoms during LC (5–8 months), mean (SD), range	1.3 (1.8) 0–10
Distribution of symptoms count during acute phase, n (%)	
None	30 (12.7%)
1–2	43 (18.2%)
3–5	93 (39.4%)
6–8	43 (18.2%)
9–12	27 (11.4%)
Distribution of symptoms count during LC (5–8 months), n (%)	
None	102 (43.2%)
1–2	106 (44.9%)
3–5	19 (8.1%)
6–8	5 (2.1%)
9–10	4 (1.7)
Persistent symptoms	
General distribution, n (%)	
None	138 (58.5%)
1 or more persistent symptom	98 (41.5%)
Range, n (%)	
1–2	86 (36.4%)
≧ 3	12 (5.1%)
New-onset symptoms, n (%)	
General distribution, n (%)	
None	167 (70.8%)
1 or more new-onset symptom	69 (29.2%)
Range of new-onset symptoms, n (%)	
1–2	55 (23.3%)
≧ 3	14 (5.9%)
Comorbidities, n (%)	
Essential hypertension	44 (18.6%)
Diabetes	21 (8.9%)
Chronic lung disorder (asthma, COPD)	17 (7.2%)
Chronic kidney disease	8 (3.4%)
Immunosuppression	7 (3%)
Heart disorder (coronary artery disease or valve disorder or heart failure)	6 (2.5%)
Neoplasia	3 (1.3%)
Solid-organ or bone marrow transplant	2 (0.8%)
No known diagnosis of chronic disorder	117 (49.6%)
COVID-19 treatment scenario (n, %)	
Non-hospitalized patients	201 (86.3%)
Hospitalized patients (COVID-19 hospital ward)	32 (13.7%)
Critical care–intensive care unit (ICU)	8 (3.4%)
Need for support therapy (n, %)	
Oxygen supplementation	24 (10.4%)
Mechanical ventilation	3 (1.3%)
Smoker, n (%)	8 (3.4%)

*WHO* World Health Organization, *LC* long COVID, *COPD* chronic obstructive pulmonary disease

^*^Missing data not computed

The mean (range) age was 41.2 (19–81), with 54.7% of individuals between 30 and 49 years old, and 144 were women (61%). BMI average (standard deviation) was 27.8 kg/m^2^ (5.7), with overweight (39.3%) and obese (27.9%) categories comprising the majority of the patients. The mean (SD, range) number of symptoms frequently reported by the patients in the acute phase of the disease was 4.1 (3.0, 0–12). Although the frequency and total number of COVID-19 typical symptoms decreased after 5–8 months to 1.3 (1.8, 0–10), a total of 98 individuals (41.5%) developed at least one symptom that persisted since acute phase (Table 1). Conversely, some symptoms were manifested only during LC, as named new-onset symptoms. They were less prevalent than persistent symptoms but affected 69 individuals (29.2%) (Table 1).

Regarding COVID-19 comorbidities, the most frequent conditions were hypertension (18.6%), diabetes (8.9%), and chronic lung disease (7.2%) as shown in Table 1.

**Fig. 1:**
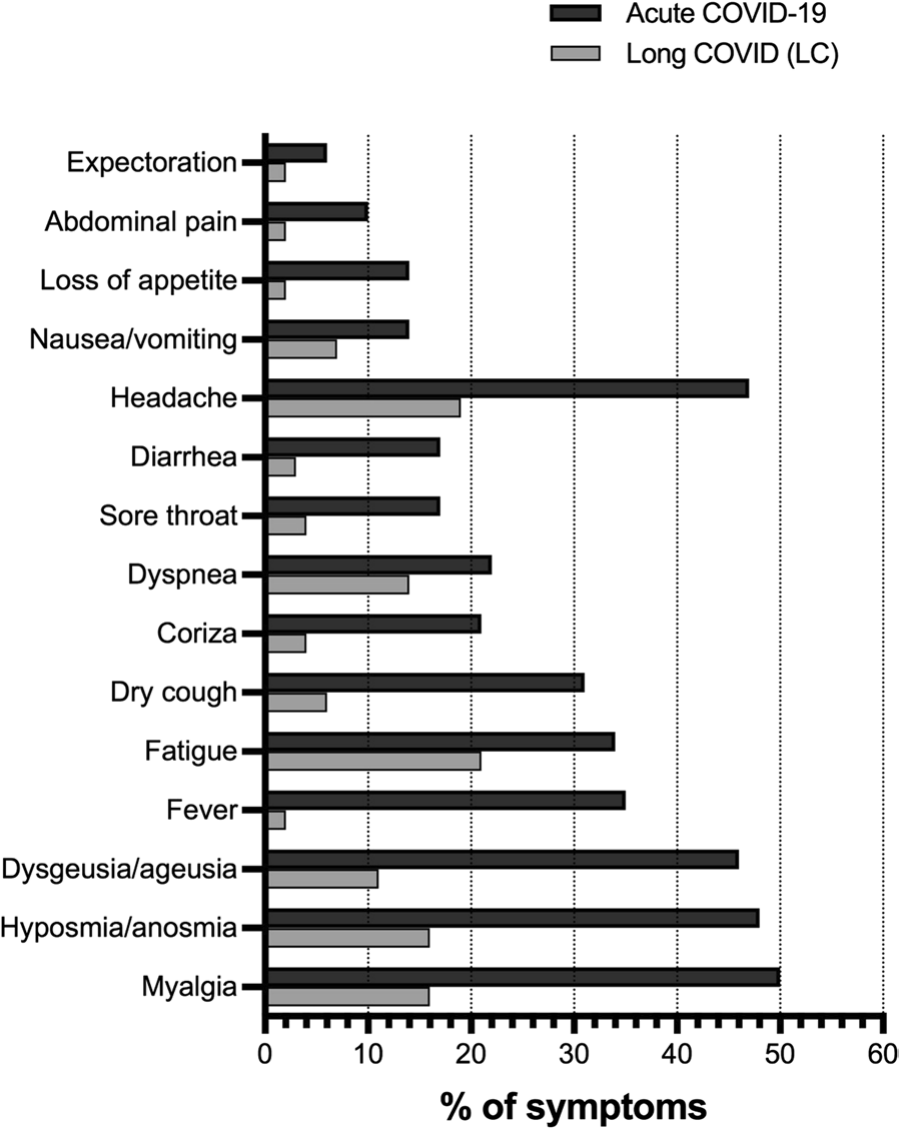
COVID-19 symptoms (percentages) during acute phase (black bars) and LC at 5–8 months after RT-qPCR positivity (gray bars) in patients from two Brazilian hospitals in mid-western Brazil. *COVID-19* coronavirus disease 2019, *LC* Long COVID

As described in Table 2, the most frequently self-reported symptoms in the acute phase were (number, %): myalgia (118, 50%), hyposmia (114, 48.3%), and dysgeusia (108, 45.8%) (also shown in Fig. 1). Fatigue was not among the three most frequent symptoms at the acute phase but affected more than one-third of individuals (80, 33.9%). In contrast, fatigue assumed the leading position during LC (5–8 m after RT-qPCR) (50, 21.2%), followed by headache (45, 19.1%) and myalgia (38, 16.1%). Fatigue is slightly more frequent as a new-onset symptom (27, 11.4%) than as a persistent one (23, 9.7%), as shown in Table 2.

**Table 2:** Typical COVID-19 symptoms frequency at acute and LC phases and respective persistence

Symptom	Phase of COVID-19 disease course		Persistence of symptoms between phases	
	Acute COVID-19^*1^, n (%)	Long COVID (LC)^*2^, n (%)	Persistent symptoms^*3^ n (%)	New-onset symptoms^*4^ n (%)
Myalgia	118 (50%)	38 (16.1%)	27 (11.4%)	11 (4.7%)
Hyposmia/anosmia	114 (48.3%)	37 (15.7%)	27 (11.4%)	10 (4.2%)
Dysgeusia/ageusia	108 (45.8%)	26 (11%)	22 (9.3%)	4 (1.7%)
Fever	83 (35.2%)	4 (1.7%)	3 (1.3%)	1 (0.4%)
Fatigue	80 (33.9%)	50 (21.2%)	23 (9.7%)	27 (11.4%)
Dry cough	74 (31.4%)	14 (5.9%)	6 (2.5%)	8 (3.4%)
Coriza	49 (20.8%)	10 (4.2%)	4 (1.7%)	6 (2.5%)
Dyspnea	51 (21.6%)	32 (13.6%)	12 (5.1%)	20 (8.5%)
Sore throat	41 (17.4%)	9 (3.8%)	5 (2.1%)	4 (1.7%)
Diarrhea	39 (16.5%)	7 (3%)	2 (0.8%)	5 (2.1%)
Headache	110 (46.6%)	45 (19.1%)	29 (12.3%)	16 (6.8%)
Nausea/vomiting	34 (14.4%)	17 (7.2%)	6 (2.5%)	11 (4.7%)
Loss of appetite	34 (14.4%)	4 (1.7%)	0 (0%)	4 (1.7%)
Abdominal pain	23 (9.7%)	4 (1.7%)	1 (0.4%)	3 (1.3%)
Expectoration	13 (5.5%)	4 (1.7%)	2 (0.8%)	2 (0.8%)

^*^^1^Period 1, acute COVID-19 phase until 14 days after positive RT-qPCR, at the first phone interview

^*2^Period 2, LC (5–8 months after RT-qPCR diagnosis), at the second follow-up phone interview

^*3^Persistent symptoms—reported at both periods 1 and 2

^*4^New-onset symptoms—reported only at period 2, e.g., not present at period 1

**Fig. 2:**
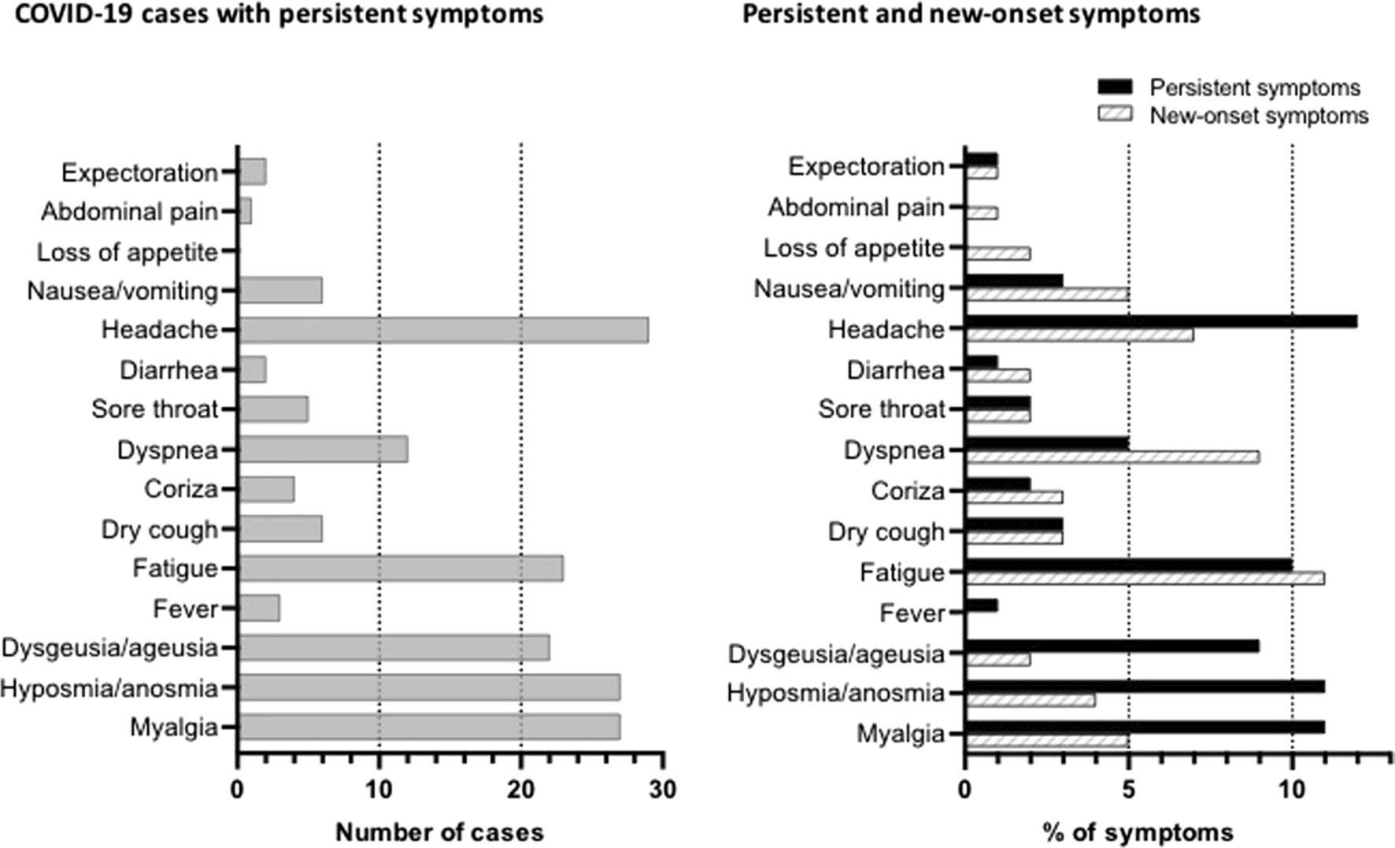
COVID-19 cases with persistent symptoms (left panel) and proportion of persistent- and new-onset symptoms (black and dashed bars, respectively, in the right panel) of LC phase in patients from two Brazilian public hospitals (n = 236). *COVID-19* coronavirus disease 2019

Among the symptoms frequently reported at LC, myalgia, hyposmia, dysgeusia, and headache were predominantly persistent and affected the following proportion of patients, 11.4%, 11.4%, 9.3%, and 12.3%, respectively. Conversely, dyspnea was mainly a new-onset symptom affecting 8.5% of individuals (the second most prevalent new-onset symptom), while fatigue affected a relatively similar proportion of individuals as a new-onset or persistent symptom (11.4% versus 9.7%) (Fig. 2, Table 2).

Our results show that SARS-CoV-2 infection leads to persistent symptoms at 5–8 months, which characterizes a chronic LC disease phase. Indeed, LC also affected relevant brain functions, caused disturbances in memory, concentration, daily activities, sleep and triggered anxious and depressive feelings.

Memory complaints were reported by 94 out of 236 individuals (39.8%) during LC, as shown in Table 3, and a relevant proportion also had problems with concentration and disability to deal with routine activities. Regarding psychiatric phenotypes, more than one-third of individuals felt anxious, and around 45% felt depressed most of the time after COVID-19. Sleep quality declined after COVID-19, with some individuals also reporting excessive daytime sleepiness, which affected 45.8% of individuals. In summary, memory complaints, sleep problems, and depressive feelings were the leading neuropsychiatric manifestations of LC in this population.

**Table 3:** New-onset neuropsychiatric symptoms frequency at the time of the follow-up phone call, 5–8 months after diagnosis, n (%) COVID phase (n = 236)

Phenotypes	Proportion, n (%)
Neurologic outcomes	
Memory complaint	94 (39.8%)
Concentration (sustained attention) complaint	73 (30.9%)
Daily activities disability	43 (18.2%)
Psychiatric and sleep complaints	
Anxiety (GAD-2 score ≥ 3 points)	87 (36.9%)
Depression/sadness (most of the time) after COVID-19	100 (42.4%)
Depression/sadness (most of the time) in the last 5 months	95 (40.3%)
Total depression (most of the time + in the last 5 months)	106 (44.9%)
Unsatisfied + very unsatisfied with sleep, n (%)	83 (35.2%)
Excessive daytime sleepiness	57 (24.2%)
Total sleep complaints: unsatisfied + very unsatisfied with sleep + excessive daytime sleepiness, n (%)	108 (45.8%)

**Fig. 3:**
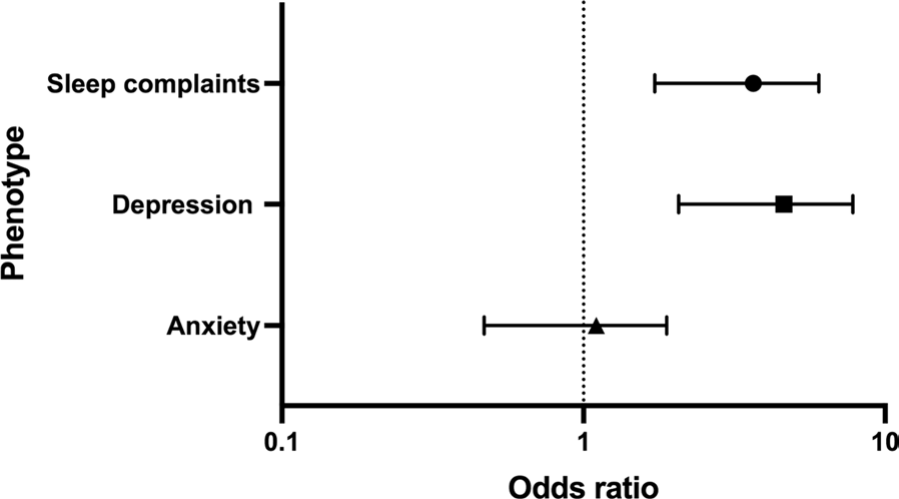
Odds ratio plot for associations between the outcome variable of memory problems and sleep, depression, and anxiety phenotypes in patients from two public hospitals of the Federal District in mid-western Brazil during 2020

We then examined whether sleep and neuropsychiatric phenotypes might have affected individuals with memory complaints to constitute a post-COVID-19 neurological syndrome. First, as shown above, memory problems have affected a substantial proportion of COVID-19 patients, as occurred for sleep complaints, depression, and anxiety phenotypes. Patients with memory problems associated with sleep complaints (n = 64) represented 27.1% of all 236 cohort individuals (Table 4). However, sleep complaints were present in 68.1% of cases of memory problems with an adjusted OR 3.206 (95% CI 1.723–6.030; *P* = 0.0003) (Table 4; Fig. 3). The same happened for depression, affecting 69.1% of memory-impaired individuals with an OR 3.981 (95% CI 2.068–7.815; *P* < 0.0001). Anxiety was also prevalent among individuals showing memory impairment but without statistical significance (Table 4; Fig. 3).

**Table 4:** Occurrence of sleep and psychiatric phenotypes in individuals with memory problems

Selected phenotypes potentially associated with memory complaints	Occurrence of phenotypes in association with memory problems regards to all positive cases (n = 236) N (%)	Proportion of cases of associated phenotypes in the subgroup of individuals referring memory problems (n = 94) N (%)	Odds ratio (95% CI)	P value
Sleep complaints	64 (27.1%)	64 (68.1%)	3.206 (1.723–6.030)***	0.0003
Depression	65 (27.5%)	65 (69.1%)	3.981 (2.068–7.815)***	< 0.0001
Anxiety	48 (20.3%)	48 (51.1%)	0.953 (0.4688–1.886)	0.8918

***p-value < 0.001

## Discussion

Knowledge about COVID-19 has rapidly evolved at an unprecedented speed thanks to a global scientific effort. This study reports on acute and persistent symptoms of RT-qPCR positive patients from hospitals in mid-western Brazil during 2020. The authors’ first concern was to verify if such a cohort would feature a proper clinical presentation for the disease in question. In general, individuals older than 60 years with comorbidities are prone to develop a more aggressive COVID-19 respiratory impairment demanding hospitalization—with 14% of them manifesting a severe form of the disease and 5% requiring critical care attention. Fortunately, the vast majority (> 80%) of patients present a mild form of the disease [2, 16]. In this study, 13.7% were hospitalized patients, 3.4% required ICU, and the remaining 86.3% were non-hospitalized individuals, thus in accordance with the literature of the symptomatic cases [2]. The age distribution of the present cohort was lower when compared to other studies with non-hospitalized patients, like in Denmark’s, The United States of America—USA’s, and the Faroe Islands’ studies [6, 7, 17]. In accordance with the present data, those studies also found that even younger individuals with favorable clinical outcomes at the acute phase may develop symptoms during the LC stage.

Regarding BMI, our study found that 67.2% of all participants were overweight or obese. Interestingly, in the study carried on in Denmark, BMI higher than normal (24.9 kg/m^2^) was a risk factor for persistent symptoms in non-hospitalized COVID-19 patients [6].

The post-COVID 19 period for collecting data was a critical point in study planning, i.e., to define the time after the acute COVID-19 enough to represent the LC. The chronic disease phase of LC includes symptoms present beyond 12 weeks after the onset of COVID-19 and is suitable for studying persistent neurological symptoms [4, 5]. Previously published articles have considered this period of 3 months before collecting data of LC; afterward, they followed individuals during a broad range of months (3–6 or more months). For example, in the Faroe Islands study, individuals diagnosed by RT-qPCR were followed after four months of disease onset [7]. In another study at a neurological COVID-19 clinic in Chicago, the mean period of analysis of post-COVID neurological symptoms was 4.72 months after symptom onset [17]. Indeed, a Chinese study also evaluated post-COVID symptoms in a period starting more than 3 months after discharge [6]. Finally, a recent systematic review on sequelae of COVID-19 included a long-term phase of 6 or more months after COVID-19 diagnoses [19].The period used in our study of 5–8 months after diagnosis thus represents the clinical presentation of chronic LC in Brazilian RT-qPCR confirmed cases.

More than half of patients in this study reported at least one symptom from 5 to 8 months after RT-qPCR positive testing, and 41.5% had at least one persistent symptom since the acute phase, including myalgia (11.4%), hyposmia (11.4%), dysgeusia (9.3%), fatigue (9.7%), and headache (12.3%). These symptoms were also prevalent in other studies with non-hospitalized patients, although at different proportions. In the Faroe Islands’ research, some persistent symptoms at 4 months post-COVID were hyposmia (24.3%), dysgeusia (16.4%), fatigue (23.9%), and headache (7.3%) [7]. In a Danish study, around 40% of non-hospitalized individuals with COVID-19 developed persistent symptoms, including fatigue (16%), smell loss (≈7.5%), taste loss (≈5%), headache (≈7%), muscle and joint pain (≈6.5%) [6]. In common, all these studies collected data more than three months after diagnosis, enrolled mainly non-hospitalized patients, and highlighted that COVID-19 leads to LC in a significant proportion of individuals with a mild form of the disease at the acute phase.

While our data strongly suggest that SARS-CoV-2 infection leads to LC, which can include typical symptoms of COVID-19, we were interested in chronic neuropsychiatric sequelae, especially sleep disturbances, anxiety, depression, and memory complaints, considered by some as part of a syndrome called ‘brain fog’ [4, 20]. In fact, few studies have yet addressed sleep complaints in LC patients [21]. “Sleep alterations” or “insomnia” can occur in up to 54% of LC outcomes [22–24], as well as daytime sleepiness as a frequent protracted symptom of acute COVID-19 [25]. In our study, sleep complaints affected 45.8% of the patients, 24.2% referring excessive daytime sleepiness, and 35.2% general dissatisfaction with their sleep quality. Not surprisingly, sleep problems were also very closely related to psychiatric symptoms as ‘sadness post-COVID’ and associated with either fear of the disease or its financial consequences [10].

There are divergences in the literature between the prevalence of post-COVID-19 depressive and anxiety disorders, probably due to the different methodologies applied. Most published studies on this subject have follow-up periods of less than three months, reduced sample sizes, lack standardization, and studies issued from a single center. Studies on previous coronavirus outbreaks show that psychiatric morbidities ranged from 10 to 35% in the post-disease stage [26], rates consistent with some studies of the current pandemic. A prospective cohort study, carried out at San Raffaele Hospital in Milan, screening for mental disorders in post-COVID-19 patients, obtained a sample of 402 patients, who were followed up for one month after hospital treatment ended. A significant proportion of them showed depression (31%) and anxiety (42%) [11]. Another multicenter observational study in Madrid, Spain, included 1200 COVID-19 hospitalized patients randomly selected from four hospitals (300 from each hospital). It was observed that seven months after admission, 16.2% had anxiety symptoms, and 19.7% had depressive symptoms [27]. In our study, we found that 36.9% of patients had anxious complaints, and 44.9% reported depressive mood after SARS-CoV-2 infection. Although mental disorders are often neglected in both acute and chronic phases of COVID-19, it is worth remembering that depressive disorders are associated with a markedly increased risk of mortality from clinical illnesses [28].

The present study identified a correlation between memory complaints and the phenotypes of sleep disturbance and sadness post-COVID. For many years a close relationship has been established between these essential physiologic functions and, more recently, sleep has been implicated in an active role in memory consolidation during rapid-eye-movement (REM) and Non-REM phases [29, 30]. Sleep is a state in which memory is optimized and consolidated, transforming short-term representations into integrated long-term ones [31]. Research shows that those findings could be valid not only for hippocampus-dependent memories but also for non-hippocampus-dependent and even for non-neuronal memories, i.e., immunological memories [31].

Another relevant point was the relationship between depression and memory complaints. It is known that cognitive impairments represent a central feature of depression [32]. Depression’s chronic stress leads to maladaptive changes that interfere with basic cognitive processing contributing to specific memory deficits [33]. On the other side, as memory troubles are distressful per se*,* they can forecast a worsening of depression [34]. Thus, the relationship between depression and memory troubles is two-fold: depression interferes with memory, and memory impairment probably exacerbates depression.

All major emergencies, including COVID-19, may cause mental health problems [35], which affect the entire population. The etiology of mental disorders is multifactorial, and the association between stress and mental health problems is determined by a variety of not only biological, but psychological and behavioral causes as well [36]. Among the risk factors for the development of sleep disorders, depression, and anxiety, we can mention concerns about changes in living conditions, pressure at work or unemployment, social isolation, financial loss, inadequate information, fear of becoming infected or infection of a family member or death, and concern about being discriminated against because of COVID-19 [35, 37–40].

On the other hand, autoimmunity or an inflammatory etiology could also contribute to possible damage to the Central Nervous System (CNS) [41], including a defective immune response that favors viral replication or a "cytokine storm" that could cause chronic physical and mental deterioration [41–43]. Elevated interleukin 6 (IL-6) levels have been associated with a poorer sleep quality in asymptomatic healthy men and women [44]. Interestingly, elevated IL-6 levels were also found in patients with acute COVID-19 [41, 45]. Therefore, this cytokine may play a role in the etiopathology of sleep disorders in the acute phase [46] and, possibly, also in the LC phase, in which sleep complaints were prevalent in the present study.

Detailing all underlying mechanisms of brain pathology caused by SARS-CoV-2 is beyond the scope of the current study. Still, it could include a hypometabolic state in important cerebral areas on the frontal (primarily orbital and olfactory areas) and temporal lobes as well as at the amygdala, hippocampus, and brainstem, in addition to neurodegeneration, gliosis, among others, that are revised elsewhere [5, 22, 26].

Finally, the present study had some limitations regarding the use of questionnaires and questions on sleep satisfaction or daytime sleepiness that could be liable to misinterpretation by the respondents. Also, the lack of a control group precludes definitive causal inferences between COVID, memory loss, and depression and could underestimate interactions with other not tested psychosocial variables during the pandemics. Better-designed evaluation tools for such neuropsychiatric variables in future studies, with the use of control groups, are warranted.

## Conclusions

The SARS-CoV-2 infection leads to persistent symptoms during LC, including fatigue, headache, and myalgia, and seems to cause neuropsychiatric sequelae. The relevant proportion of memory problems and their correlation with sleep and depressive complaints affect the quality of life and work productivity, thus requiring new research about the underlying mechanisms and therapeutics of LC and specific medical efforts to promote mental health in the pandemic and post-pandemic era.

## Supplementary Information

**Additional file 1.** Appendix 1: Questionnaire for Phase 1—Acute COVID-19; Appendix 2: Questionnaire for Phase 2—Chronic Long COVID.

## Data Availability

This current longitudinal cohort study has followed COVID-19 patients to describe the clinical picture of long COVID during months and years after disease onset. The raw data of the present work is part of this ongoing investigation and cannot be shared until the study has been completed but are available from the corresponding author on reasonable request.

## Abbreviations

BMI: Body mass index
CIs: Confidence intervals
CNPq: National Council for Scientific and Technological Development
COPD: Chronic obstructive pulmonary disease
COVID-19: Coronavirus disease 2019
HBDF: Hospital de Base do DF
HRSM: Hospital Regional de Santa Maria
ICU: Intensive care unit
IGESDF: Institute of Strategic Health Management of the Federal District
LACEN-DF: Laboratório Central de Saúde Pública do Distrito Federal
LC: Long COVID
MEC: Ministry of Education
OR: Odds ratio
SD: Standard deviation
SQL: Structured query language
REM: Rapid-eye-movement
RT-qPCR: Real-time reverse transcription polymerase chain reaction
SARS-CoV-2: Severe acute respiratory syndrome coronavirus 2
SUS: Unified Health System
USA: The United States of America
WHO: World Health Organization
